# “Topographic Shift”: a new digital approach to evaluating topographic changes of the female breast

**DOI:** 10.1007/s00404-020-05837-3

**Published:** 2020-10-20

**Authors:** Luisa Lotter, Vanessa Brébant, Andreas Eigenberger, Robin Hartmann, Karolina Mueller, Magnus Baringer, Lukas Prantl, Daniel Schiltz

**Affiliations:** 1 Department of Plastic, Hand- and Reconstructive Surgery, University Hospital Regensburg, Franz-Josef-Strauß-Allee 11, 93053 Regensburg, Germany; 2 Faculty of Mechanical Engineering, Ostbayrische Technische Hochschule Regensburg, Regensburg, Germany; 3 Center for Clinical Studies, University Hospital Regensburg, Regensburg, Germany

**Keywords:** Breast augmentation, Breast implant, 3D volumetry, 3D measurement, 3D scan, Topographic shift

## Abstract

**Purpose:**

To assess precise topographic changes of the breast, objective documentation and evaluation of pre- and postoperative results are crucial. New technologies for mapping the body using digital, three-dimensional surface measurements have offered novel ways to numerically assess the female breast. Due to the lack of clear demarcation points of the breast contour, the selection of landmarks on the breast is highly dependent on the examiner, and, therefore, is prone to error when conducting before-after comparisons of the same breast. This study describes an alternative to volumetric measurements, focusing on topographic changes of the female breast, based on three-dimensional scans.

**Method:**

The study was designed as an interventional prospective study of 10 female volunteers who had planned on having aesthetic breast augmentation with anatomical, textured implants. Three dimensional scans of the breasts were performed intraoperatively, first without and then with breast implants. The topographic change was determined as the mean distance between two three-dimensional layers before and after augmentation. This mean distance is defined as the Topographic Shift.

**Results:**

The mean implant volume was 283 cc (SD = 68.6 cc, range = 210–395 cc). The mean Topographic Shift was 7.4 mm (SD = 1.9 mm, range = 4.8–10.7 mm). The mean Topographic Shifts per quadrant were: I: 8.0 mm (SD = 3.3 mm); II: 9.2 mm (SD = 3.1 mm); III: 6.9 mm (SD = 3.5 mm); IV: 1.9 mm (SD = 4.3 mm).

**Conclusion:**

The Topographic Shift, describing the mean distance between two three-dimensional layers (for example before and after a volume changing therapy), is a new approach that can be used for assessing topographic changes of a body area. It was found that anatomical, textured breast implants cause a topographic change, particularly on the upper breast, in quadrant II, the décolleté.

## Introduction

Assessment of the precise topographic changes of the breast between pre- and postoperative states is crucial for the objective documentation of such surgery. Measurements obtained from photographs can only illustrate the topography of the breast to a limited extent [1]. Standardized photographs captured with fixed distances, angles and proportions do help to objectively document and evaluate the pre- and postoperative states of the breast [2]. However, limited technology and deviations in patient positioning as well as camera handling often provide incorrect measurements that do not allow for before-after comparisons, or the comparison between different patients.

One of the most common additional means by which numerical measurements of the female breast can be taken is the anthropometric measurement method [3, 4]. While linear and circumferential measurements methods (e.g., TTM-Chart [5]) are well described, and are established in everyday clinical practice, the three-dimensional nature of the breast can only be captured to a limited extent using these techniques. New technologies for digital three-dimensional (3D) surface body measurement have offered new possibilities to numerically assess the female breast [1, 2, 6–16]. These technologies are characterized by significantly faster data acquisition compared with linear and circumference measurements, water displacement and other conventional methods [11, 14]. In addition, 3D volumetry allows one to measure volume differences at a level of detail that is not possible with conventional two-dimensional (2D) photography, or by physical examination [17]. Evidence of a valid and reproducible analysis of the breast contour and volumetry using this method has already been proven [1, 7, 13, 17–19]. 3D volumetry also performed better in direct comparison with manual measurement methods and 2D images, and can rival the accuracy of MRI [20]. Although volumetric measurements, performed either through 3D assessment or MRI necessitate a clear demarcation of the breast contour, such anatomical demarcations have been defined inconsistently in the literature. Due to the lack of any clear demarcation of the breast contour, the annotation of landmarks which is crucial for reproducibility and reliability is highly dependent on the particular person performing the examination and therefore before-after comparisons of the same breast are error prone. The volume of the breast is also hard to define when there is no specified anatomical limit of the breast contour. Therefore, in this study, the focus is on measurement and comparison of topographic changes of the breast using 3D scans, in particular to find an alternative to volumetric measurements.

## Methods and materials

The study was designed as an interventional prospective study on 10 female volunteers between the ages of 22 and 49 years (mean 33.2 years) who were planning on undergoing aesthetic breast augmentation with anatomical, textured implants, or augmentation with subsequent mastopexy. In eight patients, the implants were placed retropectorally and in two patients epipectorally. Only anatomical, textured breast implants (Polytech, sublime line, Germany) with different volumes and bases were used in this study. 3D scans of the breasts were performed intraoperatively, without (“native”) and with (“implanted”) breast implants, after preparation of the implant pocket and temporary closure of the wounds. All scans were done in exactly the same position (55° upright position, measured by goniometer and level). The “native” and “implanted” 3D scans of the same breast allowed for precise objective comparison. The right and left breasts were considered separately from one another, as they often have substantial differences in shape and size despite belonging to the same patient. A total of 20 breasts were scanned twice (“native” and “implanted”).

All patients were treated between February and August 2019 at Caritas hospital St. Josef, Regensburg, Germany. Previous breast operations, epilepsy or breast ptosis greater than or equal to grade 3 (Regnault) were exclusion criteria. All patients were informed about the study and the potential risks. Informed consent was obtained from every patient participating in this Study. The study was approved by the Ethics Committee of the Universities of Regensburg (reference numbers: 18-885-101 and 18-1030-101). The study was planned in accordance with the Helsinki Declaration of 1975.

### 3D-Scan and measurements

A hand-held mobile 3D scanner (Eva, Artec, Luxemburg) was used to perform the intraoperative scans of the breasts. Scans were processed on a computer using Artec Studio 12 software (Artec, Luxembourg). Processing resulted in a “native” and “implanted” 3D model of each pair of breasts. Based on these, various objective evaluations could be carried out.

### Assessment of topographic changes of the breasts

**Fig. 1:**
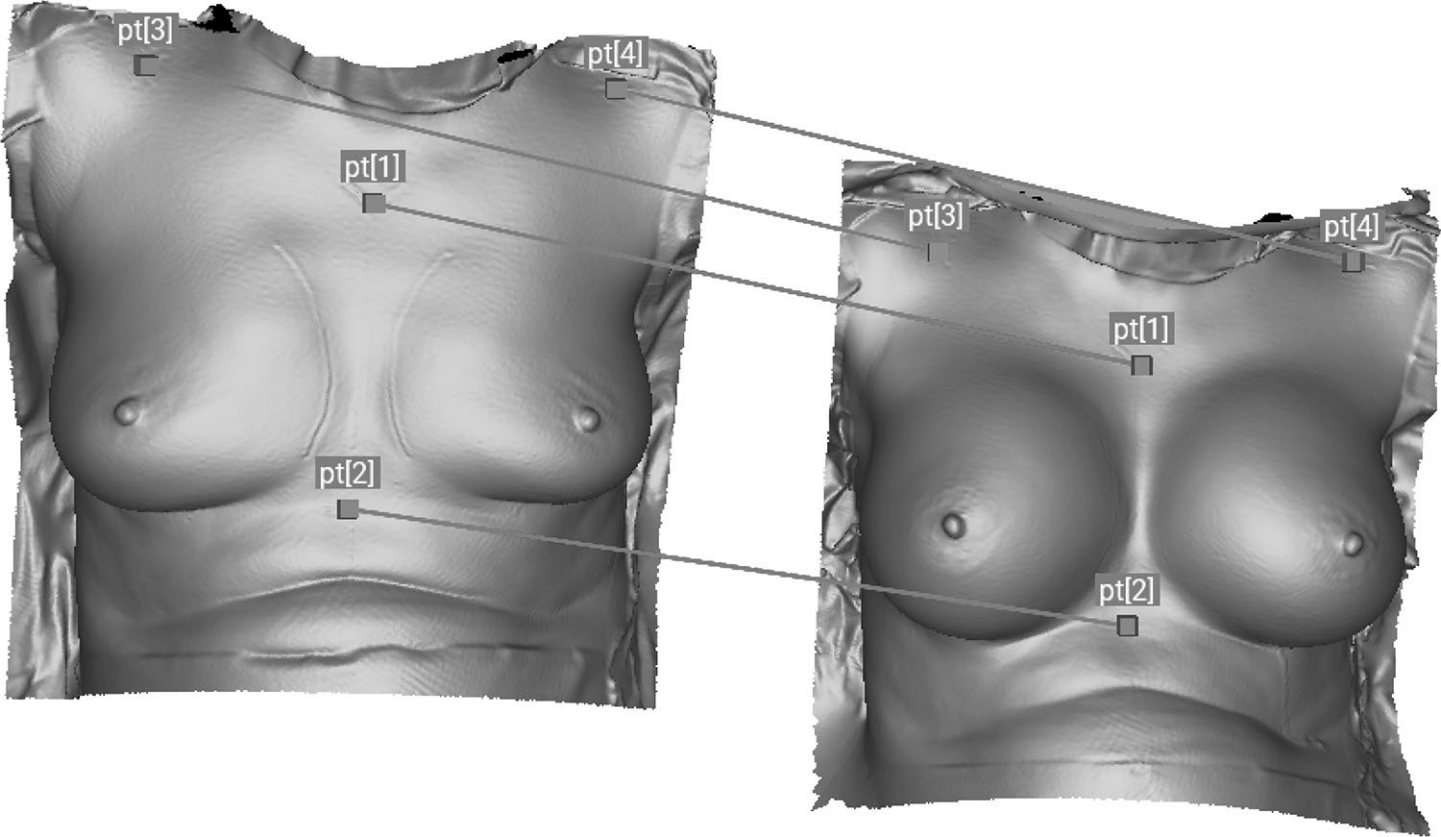
Alignment process using the anatomical landmarks. pt[1]: jugulum, pt[2]: xyphoid, pt[3]: right processus coracoideus, pt[4]: left processus coracoideus

Before topographic changes of the breast can be attributed to the insertion of the implant, the two scans of each breast must be perfectly aligned and overlaid. The “native” scan serves as a reference value (base). The Artec Studio 12 software (Artec, Luxembourg) automatically recognizes the corresponding areas and performs an alignment of both scans (“native” and “implanted”). Pre-operative markings (marked by permanent marker) on the jugulum, xyphoid and processus coracoideus served as an aid for alignment as they are fix anatomical landmarks. To guarantee that exactly the same area of the breast is measured within a patient, the scans were cut out en bloc after being perfectly aligned (Figs. 1, 3).

**Fig. 2:**
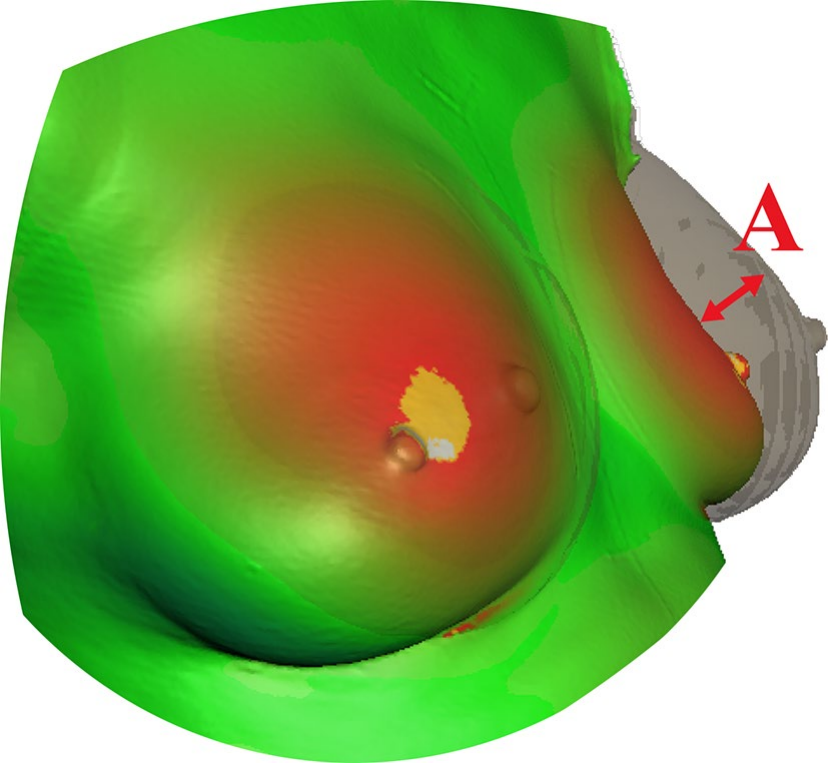
Example of aligned Scans. The Topographic Shift is the mean distance, **a** between the “native” and “implanted” scans

The software’s “measure” tool is able to automatically calculate the mean distance in millimeters (mm) between two overlaid scans. The mean distance (“topographic shift”) between the “native” scan and the “implanted” scan indicated the influence of the implants on the topography of the breast in mm (Fig. 2).

**Fig. 3:**
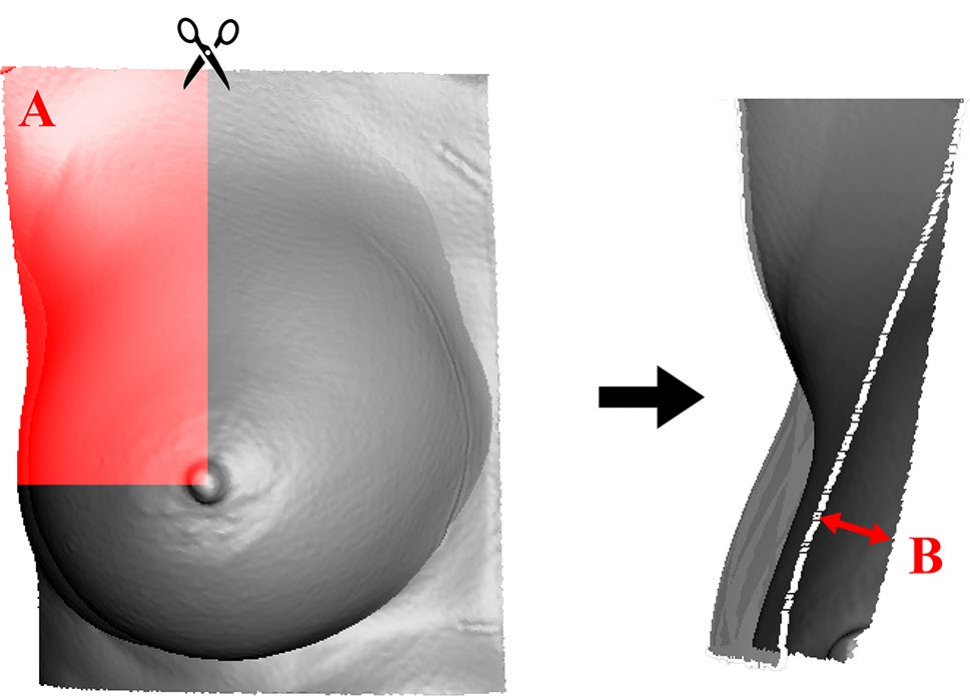
En bloc cut out of a right breast after alignment and cut out of quadrant I (red area A). The Topographic Shift of this quadrant is the mean distance, **b** between “native” and “implanted” scans

To be able to quantify the effects of the implants on the breast surface more specifically, the breast was divided into the four commonly used quadrants (quadrant I: lateral, cranial; quadrant II: medial, cranial; quadrant III: medial, caudal; quadrant IV: lateral, caudal). Quadrants were defined on the “native” scan and fixed, so that all further measurements referred to this zoning. This allowed for a more detailed analysis of the influence of the implant on the different areas of the breast. The Topographic Shift was calculated for each quadrant of each breast (Fig. 3).

## Results

**Fig. 4:**
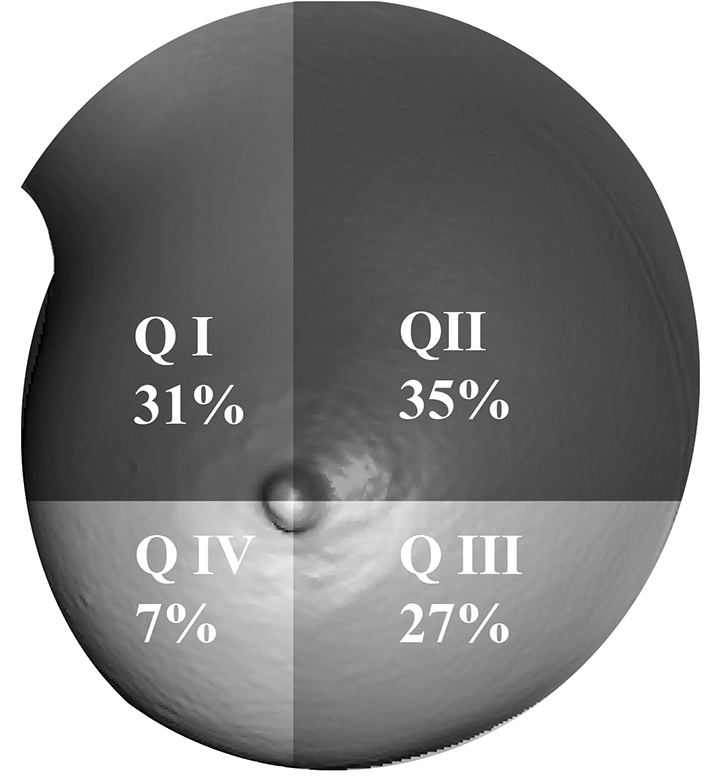
The mean distribution of total Topographic Shift in percent for all four quadrants

A total of 20 breasts were scanned twice—“native” and with “implanted”. All implants were textured, anatomical implants (Polytech, sublime line, Germany). In total, 20 implants were used. The mean implant volume was 283 cc (SD = 68.6 cc, range 210–395 cc). The mean Topographic Shift was 7.4 mm (SD = 1.9 mm, range = 4.8–10.7 mm). The mean total Topographic Shift per quadrant was: I: 8.0 mm (SD = 3.3 mm); II: 9.2 mm (SD = 3.1 mm); III: 6.9 mm (SD = 3.5 mm); IV: 1.9 mm (SD = 4.3 mm). Figure 4 shows the mean distribution in all four quadrants in percent.

## Discussion

The accuracy of 3D measurements of breasts has been proven to be accurate in several studies [1, 15, 17–19, 21], and can be considered as a precise method for objective evaluation of the breast. Koban et al. used the Artec Eva scanner to show a particularly high accuracy in the detection of even the smallest volume changes in direct comparison to analog alternatives (circumferential measurements, water displacement and others) [22]. In this study, it has been shown that the Topographic Shift is an objective measurement of topographic changes of the female breast. Results are reasonable, as breast implants ranged from 210 to 395 cc (almost double the volume) and the Topographic Shift ranged from 4.8 mm to 10.7 mm (double Topographic Shift). Furthermore, it was found that the biggest topographic change with anatomical, textured implants takes place in the upper breast, especially in quadrant II (décolleté). In quadrant IV, only small changes were measured. These results can currently not be compared to other findings, as no reports on intraoperative objective measurements of the breast could be found in the literature. Subjective comparison of round and anatomical implant shape in the same patient based on intraoperative photographs has already been performed, in which no significant difference could be detected [8, 23]. In this study, we only used anatomical, textured breast implants with different volumes and base. It would be interesting to compare the results with a cohort of round implants, or to compare different implant shapes in the same patient.

The positioning of the patient as well as the intraoperative scans were all performed by the same person to ensure a standardized measurement process. Due to the limited possibility of bringing the patient to an upright position during the operation, a maximum angle of 55° was possible without compromising the anesthesia. Intraoperative scans can therefore not be compared to pre- or postoperative scans done in an upright position. Intraoperative scans are snapshots in time that do not take into account the possible sagging of the breast over time. Eder et al. showed that one can expect a significant change in the surgical result within the first 6 months [7]. Therefore, the data presented here only provide information about the immediate influence of the implants on the breast topography, but not on long-term results.

Furthermore, it must be added that due to continuous ventilation of the patient during the scanning process the chest is in motion. If the target object moves during the recording process, the software calculates an average value (information from the manufacturer) while generating the 3D-image. Because of the calculation performed, this disruptive factor can be regarded as negligible.

It is crucial to not only scan the area to be assessed, but to extend the scan to make sure to have enough anatomical landmarks to align the scans precisely and with enough areas that are unaffected from topographic changes in the periphery, as these areas act as the base value (0 mm) for calculating the Topographic Shift. A computer is able to calculate a volume out of the Topographic Shift and most available software which can determinate volume changes can do this. However, calculations of before-after comparison are error prone, as the alignment of the scans is hard to automate. Possible reasons for this could be that the software does not recognize anatomical landmarks, the position of the scanned body area is not exactly the same in each case, or algorithms themselves may be imprecise. Furthermore, volume determination of body areas is abstract, as it is hard to define a demarcation of anatomical structures, such as the breast, cheek, abdomen, etc. The method can also be used for evaluating of pre- and postoperative scans as well. It is mandatory, that scans are done in exactly the same position.

In eight patients, the implants were placed retropectorally and epipectorally in two patients. Statistical analyses to compare these two groups was not possible, as the groups are too small. There was no difference seen in the scan results between the patients of the two groups, so that all patients were considered equally.

Cosmetic outcome is an important aspect in breast surgery. Many authors such as Camara et al. have assessed patient satisfaction after reconstructive surgery [24]. It would be interesting to discuss the different scans of the breasts with the patients and assess which implant would have come closest to their aesthetic wish. Although we see ethical concerns in confronting the patients with their scans as they might prefers other implants than the ones they finally received. A further study to analyze the aesthetic aspect of the different implants in the same patient based on the scans is planned as an online questionnaire.

3D simulations of the breast are often provided to help the patient make preoperative decisions about the implants. This should allow them to see the expected results and can improve doctor-patient communication. However, there is a lack of objective data about the effect of different breast implants on the topography of the breast [19] and the available software uses only the typical round and anatomical stigmata, such that round implants result in round breast shapes and anatomical implants results in anatomical breast shapes. Even if the match between preoperative simulations and postoperative results is high [25], the representation of the breast using outdated and falsified assumptions regarding the shape of the implant is a problem. To be able to make evidence-based predictions in the future, more intrapersonal objective evaluation is required. We think that not only the implants, but the anatomy (tissue constellation and elasticity) and especially the implant pocket are important factors influencing the final outcome. Topographic Shift at least allows for objective, numerical evaluation of topographic changes of the breast.

It has been shown that the Topographic Shift is a reasonable method for the objective measurement of topographic changes of the female breast. Measurements in this study were only performed on 20 breasts by one examiner. The method here could also be applied to other areas of the body, such as the face or the extremities. In instances where volume determination is abstract, for instance the face, the Topographic Shift could be a more concrete method to measure topographic changes after lipofilling, or other volume changing therapies.

## Conclusion

The Topographic Shift, describing the mean distance between two three-dimensional layers (for example before and after a volume changing therapy), is a new approach that can be used for assessing topographic changes of a body area. It was found that anatomical, textured breast implants cause a topographic change, particularly on the upper breast, in quadrant II, the décolleté.
